# Supplementary material for: A Reliable and Standardizable Differential PCR and qPCR Methodology Assesses HER2 Gene Amplification in Gastric Cancer
Source: Biology (Basel). 2021 Jun 10;10(6):516. doi: 10.3390/biology10060516 (PMC8230392; doi:10.3390/biology10060516)
Supplement: Supplementary file 1 [file biology-10-00516-s001.zip › biology-1228723-supplementary.pdf]

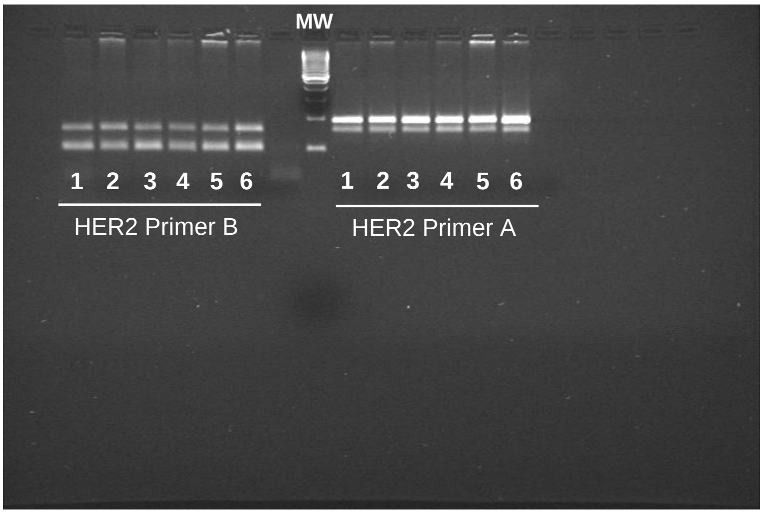

Figure S1: The The full figure of Figure 1. Samples showed in Figure 1 are the following:
Figure 1A: Samples Distal, Tumor, SKBR3/BT474 and Caco2 correspond to HER2 Primer A 1, 2, 5 and 6, respectively. Figure 1B: Samples Distal, Tumor, SKBR3/BT474 and Caco2 correspond to HER2 Primer B 2, 3, 5 and 6, respectively. MW: 100 bp ladder from 100 (lower band) to 1000. Bands size and quantification are described in Figure 1.
